# Functional and Radiological Outcome of Anterior Plate Stabilisation of the Sacroiliac Joint in Unstable Pelvic Injury

**DOI:** 10.5704/MOJ.2411.007

**Published:** 2024-11

**Authors:** MH Din, AM Aziz, Y Sahran, MA Mohamed-Saat, NS Abdul-Ghani, WI Faisham, AT Musa

**Affiliations:** 1 Department of Orthopaedics, Universiti Sains Malaysia, Kubang Kerian, Malaysia; 2 Department of Radiology, Universiti Sains Malaysia, Kubang Kerian, Malaysia

**Keywords:** pelvic ring injuries, anterior stabilisation of sacroiliac joint, functional and radiological outcome

## Abstract

**Introduction:**

Sacroiliac joint disruption, resulting from high energy trauma can cause significant morbidity if no proper treatment given. Many techniques can be used to stabilise pelvic ring injuries. We studied the functional and radiological outcome following open reduction and anterior fixation of the sacroiliac joint and agreement between both outcomes.

**Material and Methods:**

This retrospective study involved 15 patients with unstable pelvic injuries requiring surgical intervention from January 2015 to December 2020 who undergone anterior stabilisation of the sacroiliac joint. Radiological outcome assessments were done postoperatively by using Lindahl criteria. The complete functional outcome was assessed at least six months postoperatively when patients were able to weight bear by using Majeed system. Descriptive statistical analysis was performed using IBM SPSS Statistics Version 27.

**Results:**

The participants consist of 73.3% male and 26.7% female patients. A total of 66.7% of patients had a Tile type B pelvic ring injury, and the remaining 33.3% had a Tile type C pelvic ring injury. Based on the Majeed system, 73.3% of patients had excellent functional outcomes, and based on Lindahl criteria; there were 60% of patients who had excellent radiological outcome. However, there was no significant agreement between functional and radiological outcomes.

**Conclusion:**

Definitive fixation of the sacroiliac joint by anterior plate stabilisation provided an excellent functional and radiological outcome mainly due to good anatomical reduction and mechanical stability. However, further study may be needed to evaluate the correlation between functional and radiological outcomes and compare the various method of fixation with a larger sample size.

## Introduction

Pelvic fractures are infrequent injuries, accounting for about 3–8% of fractures^1,2^. Pelvic ring injury usually result from high energy trauma, for example, motor vehicle accidents and falls from height^3^. It is associated with very high mortality rate due to massive bleeding and other associated injuries^4^. According to Tilyakov *et al,* overall mortality in patients with pelvic injuries was about 9.1%. They also reported that other injuries associated with pelvic fractures were head injury, multiple fractures, and intra-abdominal injuries^5^.

Pelvic ring injury can be classified based on the stability of the posterior structures. A comprehensive AO/OTA classify pelvic ring (61) injuries into three groups. Type A (61A) is pelvic ring injury with intact posterior arch, whereas Type B (61B) refers to pelvic ring injuries with partial posterior arch disruption. In type C (61C), pelvic ring injuries are associated with complete posterior arch disruption. AO/OTA further subdivides each type into fracture group and fracture subgroup. Since there is no involvement of the pelvic ring, 61A fracture is consider as a stable fracture which involving innominate bone avulsion fracture (61A1), innominate bone fracture (61A2) and transverse fracture of sacrum and coccyx (61A3). These types of fractures can be treated non-operatively if no significant displacement. 61B fractures can be divided further into no rotational instability (61B1), rotationally unstable with unilateral posterior arch injury (61B2) and rotationally unstable with bilateral posterior arch injuries (61B3). Fractures involving 61C require surgical intervention in most of the cases because it can be rotationally and vertically unstable. In 61C1, there is unilateral posterior injury with vertical shear of the hemipelvis. In more unstable type 61C2, there is involvement of bilateral posterior injury but one of the hemipelvis is incompletely disrupted. In the most unstable type of injury (61C3), the failure is occurring through complete disruption of bilateral hemipelvis. In both B and C type, the disruption or fracture can occur either through ilium, sacroiliac joint or sacrum.

Tile classification of pelvic ring injuries consists of three major types (A, B and C). Each major type is further divided into three subtypes. Type A is a stable injury which either no ring involvement (A1), minimally displaced pelvic ring (A2) or transverse sacral fracture (A3). For type B, the pelvic ring is rotationally unstable but vertically stable. It is further subdivided into open book injury (B1), lateral compression (B2) and B3 if there is bilateral ring involvement. Pelvic ring is rotationally and vertically unstable in type C injury which can be unilateral hemipelvic (C1), bilateral hemipelvic- one side type B and the other side type C (C2) and bilateral hemipelvic with both sides type C (C3)^6^.

Tile classification has been widely used in clinical practice since it is less complicated. Furthermore, most of the literature for pelvic ring injuries also used Tile classification as their reference.

Long-term effects on unstable pelvic ring injury patients treated with non-operative treatment include lower back pain, limb-length discrepancy, neurological deficit, and possible work disability^7^. Because of these effects, the majority of patients with unstable pelvic ring injuries were treated operatively.

Many techniques can stabilise the unstable pelvic fracture, including external fixation, or internal fixation (anterior or posterior stabilisation of sacroiliac joint or percutaneous screw fixation). Therefore, good functional outcomes of the patients can be obtained if the patients undergo proper open reduction and internal fixation of the unstable posterior pelvic ring injury^8^. Unfortunately, there are no similar study assessing the radiological and functional outcome of the anteriorly stabilised sacroiliac joint. Apart from that, very limited studies found comparing the anterior stabilisation technique with other method of fixation.

Therefore, this study will evaluate radiological outcome post-operatively and functional outcome of the patients treated with anterior stabilisation of sacroiliac joint after six months surgery. We also would like to look for an agreement between those two outcomes.

## Materials and Methods

This was a cross-sectional study in a single centre, which evaluated all patients with unstable pelvic injury (Tile B and C) who underwent surgical intervention at least six months post-operatively. The fractures were also evaluated by using CT scan images with 3D reconstruction to make sure a correct classification of the fractures. The patients who were classified as Tile B1 to C3 with sacroiliac joint disruption were included in the study. Patient with sacral fracture were not included. In Tile B and C, the pelvic injuries were also involving the anterior pelvic integrity either the disruption of the symphysis pubis or superior pubic rami fracture. The included patients were treated with open reduction via ilioinguinal approach, anterior plate fixation of the sacroiliac joint and plate fixation of the anterior pelvic disruption. The operation was carried out by Advanced Trauma Team which consist of three experienced senior consultant Orthopaedic surgeons. All three of them were involved in each of the operation.

Patients with concomitant complicated injuries and complications such as traumatic brain injury with poor recovery, spine injury with permanent neurological deficit, non-union of lower limb fractures, patients with severe brain injury, bone tumours, and sacral dysmorphism were excluded.

Post-operative radiograph was obtained to assess the quality of the fixation. In our set up, no post-operative CT scan was performed due to limitation of the CT scan appointment as well as to reduce the cost of the hospital stay. A good quality plain radiograph of the pelvis in anterior-posterior (AP) view was used to evaluate the radiological outcome. Good quality pelvis radiograph was characterised by a symmetrical appearance of the obturator foramen and the iliac wing, and the distance between the symphysis pubis and the coccyx of between 1–3cm^5^. A rotated pelvis radiograph was excluded from outcome assessment.

The samples were collected from the operative census, while the primary data were collected from the patients’ files in the Medical Record Department. The samples were selected based on the inclusion and exclusion criteria, and all the samples that meet the criteria were recorded. All patients were contacted for participation. Those who agreed were asked to come for further evaluation and assessment in our Orthopaedic clinic. Radiological outcome assessment was done post-operatively, while complete functional outcome was done to the patients at least six months post-surgery as the ligaments are usually well healed after this period.

Patients were assessed based on the Majeed system for the functional outcome^9^. There were five criteria which include pain, work, sitting, sexual intercourse, and standing. Two researchers carried out the assessment, which was based on the history and physical examination. The total score from the assessment was categorised based on the grading system.

Then, the total functional score was graded as excellent if the score was more than 85, good if the score was between 70–84, fair if the score was 55–69, and poor if the score was less than 55.

**Fig. 1:**
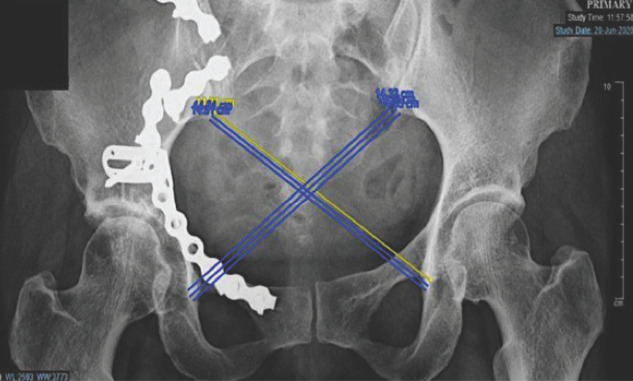
Cross measurement by Keshisyhan.

For the radiological assessment, the residual displacement of the pelvic ring was measured from the good plain radiograph of the pelvis in AP view by using the cross-measurement method of Keshishyan, which was further described by the Lefaivre^10^. The length from the inferior iliac part of the sacroiliac joint to the inferior border of the teardrop of the contralateral acetabulum was measured bilaterally (one value for length from left SI joint to the right teardrop and another value is the opposite of this). Each measurement was repeated three times, and a mean value was calculated for each length (Fig. 1). Then the residual displacement was obtained by subtracting the two values. Finally, the radiologist validated this measurement and later graded it based on 'Lindhal criteria', in which excellent was for measurement between 0–5mm, good was for measurement between 5.01–10mm, fair was for measurement between 10.0–115mm, and poor was for measurement more than 15.01mm^11^.

Data were entered and analysed using IBM SPSS Statistic Version 27. Descriptive statistics were used to summarise the socio-demographic characteristics of subjects. Based on their normality distribution, numerical data were presented as mean (SD) or median (IQR). Categorical data were presented as frequency (percentage).

For the first and second objectives, the proportion (%) of patients with excellent, good, fair, and poor outcomes were determined using descriptive statistics (estimation of proportion). The results were presented as frequency (n), proportion (%), and 95% confidence interval for a proportion. To assess agreement between the functional and radiological outcomes of the patients post-operatively, data were analysed using Cohen’s Kappa agreement. Interpretation of Cohen’s Kappa were made using recommendation, as the value of kappa between 0–0.20 is no agreement, the value of kappa between 0.21–0.39 is a minimal agreement, value of kappa between 0.40–0.59 is a weak agreement, the value of kappa between 0.60–0.79 is a moderate agreement, value of kappa between 0.80–0.90 is a strong agreement, and value of kappa more than 0.90 is almost perfect agreement^12^.

## Results

A total of 20 patients with unstable pelvic ring injury were operated on with anterior stabilisation of sacroiliac join fixation from January 2015 until December 2020. Five patients were excluded from this study: one patient had poor Glasgow Coma Scale recovery secondary to traumatic brain injury, one patient had an ischemic stroke, and the other three had rotated view of pelvis plain radiograph. Thus, a total of 15 participants were recruited for this study. The mean age was 30.80 years (SD=12.13). There were more than half male participants (n=11, 73.3%) among the total samples recruited. For Tile Classification, 66.7% of patients had type B pelvic injury, and 33.3% had type C pelvic injury. In addition, all patients had other concomitant injuries such as brain injuries, intraabdominal injuries, acetabulum fractures, and limb fractures. However, all of these injuries were managed accordingly, and none of the patients had residual complications or morbidity pertaining to the injuries they sustained (Table I).

**Table I:** Demographic data of study sample (n=15).

Variables	Total n (%)
Age, years (mean, SD)	30.80 (12.13)
Gender	
Male	11 (73.3)
Female	4 (26.7)
Type pelvic injury	
Type B	10 (66.7)
Type C	5 (33.3)
Concomitant injury	
Yes	15 (100.0)
No	0 (0.0)
Post-op complication	
Yes	4 (26.7)
No	11 (73.3)

For the post-operative complications, three patients had a superficial surgical site infection. However, all the infected wounds were managed surgically by wound debridement, and patients also were given adequate antibiotics. Eventually, all the wounds have healed. One patient developed avascular necrosis of the femoral head likely due to concomitant acetabular fractures and hip dislocation. However, our assessment revealed that this patient had a good functional outcome. This patient had pain during moderate activity and had very limited time and walking distance due to pain. Other than that, none of our patients had any neurological deficit post-operatively. Table II showed the summary of the patients based on the Tile Classification and their respective functional and radiological outcomes.

**Table II:** List of patients participated in this study.

Patients’ ID	Tile Classification	Functional outcome	Radiological outcome (mm)
1	Tile C	82 (Good)	12.6 (Fair)
2	Tile C	100 (Excellent)	3.5 (Excellent)
3	Tile B	95 (Excellent)	8.9 (Good)
4	Tile B	86 (Excellent)	1.5 (Excellent)
5	Tile B	75 (Good)	14.2 (Fair)
6	Tile B	100 (Excellent)	6 (Good)
7	Tile C	96 (Excellent)	1.5 (Excellent)
8	Tile C	100 (Excellent)	0.2 (Excellent)
9	Tile B	76 (Good)	2.1 (Excellent)
10	Tile B	100 (Excellent)	0.3 (Excellent)
11	Tile B	100 (Excellent)	4.7 (Excellent)
12	Tile C	73 (Good)	13.8 (Fair)
13	Tile B	100 (Excellent)	4.2 (Excellent)
14	Tile B	100 (Excellent)	0.9 (Excellent)
15	Tile B	100 (Excellent)	7.6 (Good)

For the functional outcome of the patients with unstable pelvic ring injuries treated with anterior stabilisation of SIJ, 73.3% (n=11) of the patient fall in the excellent group. The mean total score of the functional outcome was 92.20 (SD=10.62). The summary of the functional result is presented in Table III.

**Table III:** Functional outcome based on grade (n=15).

Variable	Mean (SD)
Pain	27.00 (4.93)
Work	18.40 (2.53)
Sitting	9.60 (1.12)
Sexual intercourse	4.00 (0.00)
Standing walking aided	11.87 (0.52)
Standing gait unaided	11.07 (1.67)
Standing walking distance	10.27 (3.01)
Total score functional outcome	92.20 (10.62)
Functional outcome, n (%)	
Excellent	11 (73.3)
Good	4 (26.7)
Fair	0 (0.0)

The distribution of the radiological outcome of participants is presented in Table IV. A total of nine patients (60%) falls in the excellent group. Other patients were in the good and fair groups, and none were found in the poor group.

**Table IV:** Radiological outcome based on grade (n=15).

Variable	n (%)
Residual displacement	5.47 (4.91)
Radiological outcome	
Excellent	9 (60.0)
Good	3 (20.0)
Fair	3 (20.0)

The agreement between radiological outcome and functional outcome was summarised in Table V. The Kappa test showed no significant agreement between radiological and functional outcomes (p=0.660). Radiological outcomes reported three excellent, good, and fair groups, while functional outcomes have none for the fair group.

**Table V:** Agreement between functional and radiological outcome of anterior SIJ stabilisation (n=15).

Variables	Radiological outcome		Fair n (%)
	Excellent n (%)	Good n (%)	
Functional outcome			
Excellent	8 (53.3)	3 (20.0)	0 (0.0)
Good	1 (6.7)	0 (0.0)	3 (20.0)
Agreement (Kappa)	0.08 (p=0.660)		

## Discussion

Even though pelvic ring injuries are rare, orthopaedic surgeons may have difficulties managing such injuries, especially unstable pelvic ring injuries. In addition, the patients may come with some complex injuries to the pelvic ring and are usually associated with other concomitant injuries resulting from high energy trauma, thus leading to life-threatening conditions. Therefore, initial management of rapid and prompt clinical and radiological assessments, including plain radiograph and CT-scan, is mandatory before deciding for stabilisation of the pelvic ring to maintain the hemodynamic stability^13,14^.

Significant disabilities may occur in patients with unstable pelvic ring injuries in a long-term period if these injuries are not treated properly. Richard C. Henderson found out that patients may have chronic pain, limb length discrepancy, poor working performance, and neurological deficit if left untreated^7^. The study concluded that the low back pain in patients with unstable pelvic injury had a significant correlation with the residual displacement. This will lead to work-related disabilities. It was also mentioned that limb length discrepancy is mainly associated with pelvic obliquity. Still, other factors such as pain, weakness, or other lower limbs injuries may affect the discrepancy. On the other hand, certain patients may have permanent neurological deficit includes weakness, numbness, or burning sensation of the lower extremities, depending on residual displacement. Apart from the prevention of the neurological injury, the aim of the fixation in this type of injury is to get an equal limb length, achieve a symmetry pelvis and prevent displacement of the fracture or dislocation.

Many techniques can stabilise the unstable pelvic fracture, including external fixation or internal fixation (anterior or posterior stabilisation of SIJ or percutaneous screw fixation)^8,15-17^. Pelvic external fixation can be used for emergency treatment for unstable pelvic ring injury as it reduces and maintains the volume, thus improving the tamponade and reducing the bleeding^11^. However, Tile *et al* mentioned that the pelvic external fixator could not fully stabilise the pelvic ring injury and biomechanically is inferior to the other treatment method; thus, this technique is rarely used as the definitive treatment^18^.

Posterior transiliac plating is also one of the surgical techniques that can stabilise the unstable pelvic injury, especially the vertical type of injury. Suzuki *et al* did a study on 19 patients treated with this fixation. He concluded that the mean Majeed functional assessment score was 78.5 (good functional outcome). However, due to the injury that can cause Morel-Lavallee lesion, the patients will be highly susceptible to wound breakdown post-operatively. Thus, he suggests that soft tissue conditions must be evaluated thoroughly before choosing this type of fixation^15^.

As for current practice, most orthopaedic surgeons prefer to do definitive fixation by doing the percutaneous sacroiliac screw fixation. It is because it reduces wound complications and bleeding and does not interrupt pelvic hematoma. However, this percutaneous technique is unable to achieve a good anatomical reduction due to the limited method for reduction. As a result, this will affect the functional outcome of the patients^16^. Matta also explained that the surgeon must understand the anatomy in terms of bone, vascular, and neurological structures of the posterior pelvis^17^. Schweitzer *et al* had found that there are a few patients who had complications related to this technique, such as nerve root injury and screw misplacement^16^. They found out that 1% of the patients had S1 nerve root compression, in which they needed to do surgical decompression and screw removal. In terms of screw misplacement, they noted that 3% of the patients had such complications. In order to get a good reduction of the sacroiliac joint, the surgeons usually utilised the lateral window of the ilioinguinal approach before inserting the sacroiliac screws. This method reduces the incidence of malreduction of the sacroiliac joint by using the percutaneous screws alone, thus reducing the incidence of malreduction, injury to the nerve root and screw misplacement.

In our practice, we chose anterior stabilisation of the sacroiliac joint with plating as a definitive fixation of unstable pelvic ring injuries. This surgical technique allows us to visualise the joint, remove all the soft tissue interposition particularly the inverted ruptured ligament and capsule, and bony fracture that prevent us from getting a good reduction. By using this approach, we were able to achieve good anatomical reduction of the sacroiliac joint^8^. Leighton *et al* through his biomechanical study, showed anterior stabilisation of the sacroiliac joint with plating has better biomechanical fixation than sacroiliac screw fixation^19^. Therefore, we used the anterior ilioinguinal approach in all of our patients. However, we used some modification of the lateral window. Instead of elevating the iliacus, we used the interval between the iliopsoas muscles and femoral vasculature (artery and vein) to reach the sacroiliac joint. By doing this, we can have a better visualisation of the joint and a better trajectory of the drill bit for screw insertion. We were also able to visualise the nerve root of L5 and protect it during the procedure. Our study demonstrated no patient with neurological complication. Having direct visualisation around the sacroiliac joint, we could prevent injury to the surrounding neurovascular structures and able to control the haemostasis well. In most of the cases, three transverse crossing arteries over the sacroiliac joint were torn due to the impact of the sacroiliac joint separation but still we have to ligate it to secure the bleeding intra-operatively. The other consistent source of the bleeding with this approach is the nutrient artery coming from the iliac bone about 1cm lateral to the inferior part of the sacroiliac joint which require bone wax to stop the bleeding in most of the cases.

To reduce the dislocation, we inserted one 3.5mm cortical screw at the iliac brim and another screw at the brim of the sacrum, less than 1cm medial from the joint, to prevent injury to the L5 nerve root. We choose the centre region of the sacroiliac joint for screw insertion to get an equal force during reduction process. Then, the reduction was achieved by using the Jungbluth clamp, and the joint was fixed with two 3-hole plates with 80° to 90° orientation to each other to achieve maximum mechanical stability. This clamp can be used to correct both the vertical and anteroposterior displacement and we found it so useful. However, the reduction must be done carefully to make sure no breakage of the iliac or sacral brim with excessive force of the clamp. Sometimes 2mm threaded pin can be used as a joystick to help achieve a good reduction of the dislocation. The plates position must be well planned. Most important position of the plate is at the inferior brim of the sacroiliac joint because we can get a better purchase of the screw due to good quality of the bone at that region. The other plate can be positioned at the centre part of the sacroiliac joint. This good stability of the construct is important to prevent the vertical displacement of the hemipelvis especially during weight bearing. Even though this approach was technically demanding, we believe that the anatomical reduction of the sacroiliac joint is the main contributing factor for the excellent outcome.

**Fig. 2:**
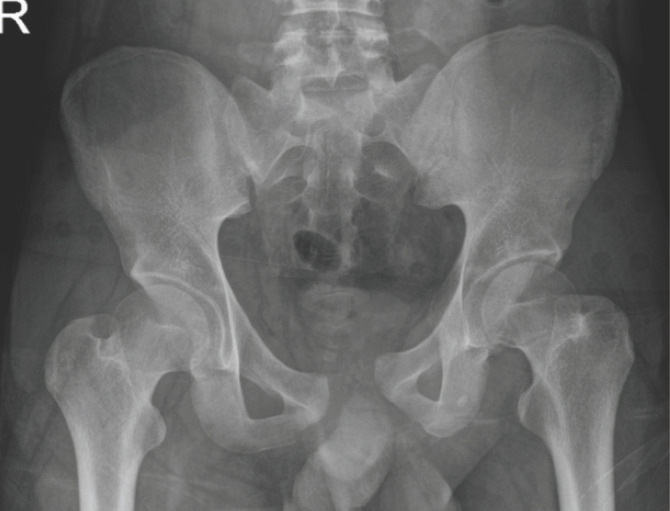
Pre-operative pelvic radiograph.

**Fig. 3:**
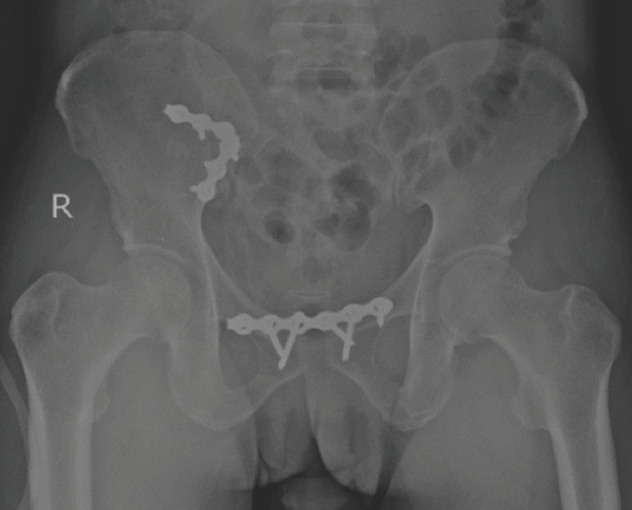
Post-operative pelvic radiograph.

Open reduction allows us to visualise the joint directly; thus, excellent reduction can be achieved (Fig. 2 and Fig. 3). In our study, 12 out of 15 patients had an excellent and good residual displacement (less than 5mm), accounting for about 80% of our patients. According to Matta and Tornetta, about 95% of patients had an excellent and good residual displacement of the joint after open reduction^20^. Thus, despite differences in methodology, our radiological assessment result is similar to the previous study.

Numerous studies have shown excellent and good result functional outcomes on patients with pelvic injuries treated with internal fixation regardless of the technique. Pohlemann *et al* had shown that 79% of patients had an excellent and good functional outcome in Tile type-B pelvic ring injury. However, only 27% of patients with Tile type-C pelvic ring injury had excellent and good results despite the fractures healed anatomically and having less than 5mm residual displacement^2^. Another study done by Lindahl and Hirvensalo showed 83% of patients had an excellent and good functional outcome. They concluded that a few factors could contribute to the unsatisfactory functional outcome, such as poor reduction, failure of fixation, and permanent lumbosacral plexus injury^21^.

As for our study, 73.3% of the patients had an excellent functional outcome post-operatively. However, we found out that four patients had a good functional outcome. Two of these patients had complications post-operatively; one had surgical site infection, and another had avascular necrosis of the femoral head in a patient with an associated acetabular fracture and hip dislocation. The other two patients had a good functional outcome with a fair radiological outcome score; these patients had mechanical pain with very limited time and distance of walking. Thus, the possibility of inadequate reduction of sacroiliac joint may affect the patients’ functional outcome.

Functional outcome of patients in unstable pelvic injury treated with internal fixation had closely related with radiological outcome post-operatively. Further evaluation by Lindahl and Hirvensalo stated a strong association between excellent radiological outcome and functional outcome^21^. Even though our study shows no significant agreement between functional and radiological outcomes (p-value <0.05), about 53.3% of patients had excellent functional and radiological outcomes, we postulated that these results could be due to the small number of patients we gathered in this study. On the other hand, we noticed that three patients had fair radiological outcomes but good functional outcomes. These results could be due to the excellent rehabilitation program that these patients went through post-operatively. Rehabilitation programs such as physiotherapy with good pain control management may improve the quality of life, thus may give great functional outcomes.

We had encountered a few limitations in this study. Small numbers of samples in this study have a significant effect on the result of this study in terms of agreement and correlation between functional and radiological outcomes. The number of patients with an unstable pelvic injury that has undergone anterior stabilisation in our centre is average around five cases per year. Other than that, HUSM is the only centre that solely performs this fixation in unstable pelvic injury compared to other centres in Malaysia. Difficulties in getting the patients’ records from the previous year’s add to the limitation.

Besides, there are no previous study or research that was done specifically on this type of surgical fixation related to functional and radiological outcomes. In the previous research involving patients treated with all types of fixations, the numbers of the patients were also very limited, as it is not a commonly done procedure. Thus, we suggest that large numbers of samples are needed by doing the study in multiple centres with good access to patients’ records; therefore, it will give a better and more significant result. Other than that, a comparison of the outcome of different types of techniques also can be made.

The availability of radiographs also plays a vital role in evaluating radiological outcomes. Therefore, we suggest that every patient should undergo complete plain radiograph of the pelvis (anterior-posterior view, inlet view, and outlet view) as well as a CT scan post-operatively so that a better assessment can be done. We have to exclude a few patients due to improperly taken post-operative radiograph. This significantly contributed to small number of samples in our study. Post-operative CT scan evaluation will be the best method to assess the radiological outcome.

## Conclusion

Unstable pelvic ring injuries require a rapid and prompt evaluation to prevent the life-threatening condition and decide on the definitive management of the patients. The aims of the operative intervention are to prevent neurological injury, to achieve good anatomical reduction and provide a good stability of the construct thus restoring the pelvis symmetry and limb length equality. We believe that anterior stabilisation of the sacroiliac joint can give good anatomical reduction with superior mechanical stability and excellent functional and radiological outcomes with a lower incidence of complications. However, further study is needed with a larger number of samples and good radiological assessment to compare with other methods of fixation.
